# Editorial: Nutraceuticals in cardiovascular diseases and their associated risk conditions

**DOI:** 10.3389/fcvm.2024.1468355

**Published:** 2024-09-04

**Authors:** Lamiaa Ahmed, Naufal Zagidullin

**Affiliations:** ^1^ Department of Pharmacology and Toxicology, Faculty of Pharmacy, Cairo University, Cairo; ^2^ Department of Internal Diseases, Bashkir State Medical University, Ufa, Russia

**Keywords:** cardiovascular diseases, inflammation, nutraceuticals, oxidative stress, comorbidity, cardioprotection

**Editorial on the Research Topic**
Nutraceuticals in cardiovascular diseases and their associated risk conditions

Cardiovascular diseases and their associated risk conditions including dyslipidemia, hypertension, coronary heart disease and diabetes are one of the main health issues worldwide. The mechanisms underlying cardiovascular disorders are complex and multifactorial including oxidative stress, inflammation and mitochondrial dysfunction as well as modulating the activities of several kinases and phosphatases. Importantly, pharmacotherapies available for the management of these diseases are considered insufficient and show several limitations and side effects especially in high-risk patients.

Nutraceuticals are bioactive food components or phytochemicals that provide benefits including the prevention or treatment of several diseases. Nutraceuticals such as flavonoids, vitamins and other natural substances have shown pleiotropic antioxidant and anti-inflammatory properties. These natural components enriched in dietary substances are believed to target multiple pathways in slow and effective ways without causing significant side effects. Current evidence potentiates the application of nutraceuticals to enhance the efficacy of pharmacological therapies and to reduce their adverse effects. A number of studies have shown that cardiovascular diseases are strongly correlated with dietary habits of patients. However, limited studies are available about the use nutraceuticals in cardiovascular diseases, their long-term safety and effectiveness especially in clinical trials and thus more research is required in this direction. This issue was designed to shed more light on the use of nutraceuticals for the management of cardiovascular diseases and their associated risk conditions with emphasis on their protective potentials in both experimental and clinical studies and the underlying mechanisms.

Hyperactivation of blood platelets leads to various thrombotic events, including embolies, thrombotic stroke, etc., and could be influenced by various dietary components from vegetables, fruits, teas, wines, cocoa and its products. The cocoa products including chocolate and coffee are very widespread in the world and their cardioprotective function and interaction with other drugs regarding hypercoagulation and bleeding are of special interest. Olas examined the impact cocoa and its products, including chocolate, on the number and function of thrombocytes, and the anti-platelet activity of their phenolic compounds. In conclusion, it was shown that cocoa consumption, especially in the form of dark chocolate, mostly due to the high flavanol content, had anti-platelet activity and might play a significant role in cardioprotection. Thus, the high cocoa products consumption may be an excellent strategy in primary prevention of cardiovascular events through decreasing cardiovascular risk, including hyperactivation of thrombocytes.

Coronary and systemic atherosclerosis is a significant cause of cardiovascular and cerebrovascular diseases, with a greater impact on men than women. Wang and Wang investigated the association between dietary antioxidants intake and the ankle brachial pressure index (ABPI), the marker of arterial compliance and endothelial dysfunction which could be also used for assessing the progression of atherosclerosis. In this cross-sectional designed study with more than thousand participants from the NHANES survey (USA), the six antioxidants (selenium, carotenoids, zinc, and vitamins A, C, E) and a composite dietary antioxidant index (CDAI) were investigated as risk factors of ABPI. The multiple regression analysis revealed that among men but not women, the dietary intake of selenium, zinc, and vitamin A was positively associated with a higher ABPI and between CDAI and ABPI, a U-shaped association was presented. The important issue was also that CDAI, high vitamin A, C, and E intake had a direct effect on all-cause mortality. In this regard, there was a significant interaction between high intake of vitamin A and gender, with a superior effect for women. As a result, the intake of dietary nutrients with antioxidant properties may prevent vascular atherosclerosis and improve vascular compliance in a sex-dependent manner.

Carnosine is an antioxidant and an endogenous dipeptide that has been widely assessed as a protective agent in various studies. A study by Berdaweel et al. revealed the antifibrotic potential of L-carnosine and its ability to mitigate the adverse cardiac remodeling in an experimental mouse model that mimics pathophysiological basis of type II diabetes and metabolic diseases in patients. Either wild type or GPx4+/−mice were exposed to high sucrose high fat diet (HSHF) for 16 weeks. Carnosine treatment moderately improved glycemic control with non-significant effect on insulin resistance. Interestingly, carnosine significantly upregulated cardiac GPx4 expression and reduced levels of iron and protein carbonyls in all genotypes exposed to HSHF diet. Furthermore, *in vitro* experiments demonstrated the carnosine's ability to inhibit crosslinking of collagen. In summary, this research provides a valuable information regarding the antifibrotic potential of carnosine and the possible underlying mechanisms.

Cafestol is a natural diterpene that presents in coffee. It shows distinctive anti-inflammatory and antioxidant in addition to antimutagenic properties in various investigations. A study by Al-Kenany and Al-Shawi provided data about the obvious protective effect of cafestol in cardiotoxicity induced by doxorubicin in rats. Cafestol significantly improved cardiac injury induced by doxorubicin and improved histopathological changes through inhibition of cardiac oxidative stress and enhancement of Nrf2 expression and the associated downstream antioxidant genes. Moreover, cafestol significantly inhibited inflammatory markers (IL-1β and TNF-α) and cardiac apoptosis (TUNEL-positive cardiomyocytes, Bax, and Casp 3). These outcomes reveal the potential of cafestol use as an adjuvant to chemotherapy to alleviate doxorubicin-induced toxicities.

Therefore, the current special issue revealed the safety and efficacy of nutraceuticals such as cocoa products, dietary composite antioxidants, carnosine, cafestol, etc. in various cardiovascular diseases. We aim that this research will motivate more experimental and clinical studies, providing new directions for the early prevention and management of various cardiovascular conditions.

